# Folic acid supplementation ameliorates alcohol-induced hepatic steatosis by inhibiting SREBP-1c-mediated lipogenesis

**DOI:** 10.3389/fnut.2025.1668430

**Published:** 2025-09-24

**Authors:** Chen Liang, Hao Sun, Tongtong Lan, Junlei Yin, Huichao Zhao

**Affiliations:** ^1^Qingdao Hospital, University of Health and Rehabilitation Sciences (Qingdao Municipal Hospital), Qingdao, China; ^2^College of Medicine, Qingdao University, Qingdao, China

**Keywords:** alcoholic fatty liver disease, folic acid, 5-MTHF, lipogenesis, SREBP-1c

## Abstract

**Background:**

Alcoholic fatty liver disease (AFLD), a prevalent yet reversible stage of alcoholic liver pathology, is often associated with folate deficiency. This study investigated the association between folate status and AFLD risk and explored the underlying mechanisms.

**Methods:**

Data from NHANES 2011–2020 (*n* = 10,452; 259 with AFLD) were analyzed. Associations between dietary folate equivalent (DFE), serum folate, 5-methyltetrahydrofolate (5-MTHF), red blood cell (RBC) folate, and AFLD were evaluated using multivariable logistic regression, adjusting for demographic and clinical variables. In parallel, ethanol-fed C57BL/6J mice, with or without folic acid supplementation, and L02 hepatocyte models were used to assess biochemical markers, hepatic histology, and lipogenesis-related protein expression.

**Results:**

Higher serum folate levels were significantly associated with reduced AFLD risk across all adjusted models (Model 3 Q4 vs. Q1, OR = 0.35, 95% CI: 0.22–0.54). Serum 5-MTHF levels were inversely associated with the severity of steatosis (*p* < 0.01). In contrast, elevated RBC folate was a risk factor in specific subgroups. Folic acid intervention *in vivo/in vitro* reduced ethanol-induced increases in hepatic enzymes, TG, hepatic lipid accumulation, and expression of lipogenic proteins (SREBP-1c, FASN, ACC1; *p* < 0.05), but not SCD-1.

**Conclusion:**

Serum folate and 5-MTHF are protective factors against AFLD. Furthermore, folic acid can ameliorate hepatic steatosis by inhibiting SREBP-1c-mediated lipogenesis, highlighting its potential in AFLD prevention and therapy.

## 1 Introduction

Alcoholic fatty liver disease (AFLD), the initial stage of alcoholic liver disease (ALD), represents a significant global health concern due to its high prevalence and potential reversibility (1). Prompt intervention is essential to enhance liver function and prevent progression of ALD to more advanced stages, including alcoholic hepatitis, liver cirrhosis, and hepatocellular carcinoma. According to WHO reports, approximately 3.3 million deaths worldwide each year are attributed to alcohol abuse, accounting for 5.3% of all global deaths (2). In China, the incidence of ALD has been rising annually. The proportion of ALD patients among hospitalized liver disease cases increased from 2.95% in 2005 to 6.84% in 2019 (3). With the growing consumption of alcohol, AFLD has emerged as a major public health concern.

Alcohol induces persistent and progressive hepatocellular damage through an integrated network involving oxidative stress, gut–liver axis-mediated inflammatory reprogramming, and both genetic and dynamic epigenetic modifications. These mechanisms operate within a multi-systemic crosstalk between the gastrointestinal tract and the innate and adaptive immune systems. Although therapeutic breakthroughs have substantially improved in chronic viral hepatitis, metabolic-associated fatty liver disease (MASLD), and autoimmune liver diseases (4, 5). Currently, beyond abstinence, treatment options for late-stage ALD, such as steroids and liver transplantation, are available. However, effective treatments and early interventions remain limited and are usually associated with side effects, including increased risks of infection and osteoporosis (3). As a result, current ALD guidelines emphasize nutritional support, specifically recommending supplementation with B vitamins and other micronutrients (6). This emphasis is crucial, as alcohol-associated liver disease, particularly in its initial phases such as AFLD, compromises the liver’s capacity for nutrient processing and storage. Therapeutic B-vitamin supplementation directly counters these nutritional deficits and facilitates the restoration of optimal hepatic functions.

Folic acid, a synthetic folate commonly used as a dietary supplement, is converted in the body to its bioactive form, 5-methyltetrahydrofolate (5-MTHF). Serum folate, which predominantly consists of 5-MTHF, reflects recent dietary intake and is widely used to assess short-term folate status (7). In contrast, red blood cell (RBC) folate represents folate incorporated during erythropoiesis and serves as a marker of long-term folate stores (8). As a one-carbon carrier, 5-MTHF participates in essential cellular processes, including nucleic acid and neurotransmitter synthesis, as well as homocysteine (Hcy) remethylation (9). Adequate levels of both serum and RBC folate are critical for maintaining folate-dependent metabolic functions. Epidemiological data show that approximately 69–80% of alcohol abusers have serum folate deficiency, which can lead to megaloblastic anemia in severe cases (8). Long-term alcohol consumption can affect folate metabolism by reducing its uptake, storage, and reabsorption (10). It can also impair folate conversion and utilization, thereby altering the overall DNA methylation level in the liver (11).

Exogenous folic acid supplementation has a protective effect against alcohol-induced liver damage. Animal experiments have shown that folic acid intervention can reduce the increase in serum hepatic enzymes and oxidative stress levels caused by alcohol exposure, and alleviate liver damage (12). Previous research in our laboratory has demonstrated that folic acid intervention can alleviate the hepatic endoplasmic reticulum stress levels of ALD mice and improve mitochondrial function (12, 13). Moreover, folic acid can influence the composition of the gut microbiota, thereby affecting the gut–liver axis and ameliorating hepatic inflammation (14). These findings collectively suggest that folic acid could serve as a key nutrient for the improvement of ALD. In non-alcoholic fatty liver disease (NAFLD) animal models, folic acid can regulate the PI3K-AKT-SREBP pathway by influencing hepatic insulin/IGF2 signaling, thereby reducing liver lipid accumulation (15). Additionally, adjusting the diet of NAFLD patients to increase the abundance of folate-producing microbiota can raise circulating folate levels. This, in turn, downregulates hepatic fatty acid synthesis signaling, ameliorates hepatic insulin resistance, and reduces abnormal lipid accumulation (16). However, it is not yet known whether folic acid supplementation can regulate alcohol-induced hepatic lipid metabolism and improve hepatic steatosis.

In this study, we used data from the NHANES 2011–2020 epidemiological survey, supported by animal and cell experiments, to investigate the efficacy of folate in alleviating alcoholic hepatic steatosis and to preliminarily explore its underlying mechanisms.

## 2 Materials and methods

### 2.1 Data source

This study used data from the National Health and Nutrition Examination Survey (NHANES online: https://www.cdc.gov/nchs/nhanes/index.html), spanning the years 2011 to 2020. The dataset includes demographic information, anthropometric measurements, laboratory tests, and alcohol intake questionnaire (ALQ). All procedures were approved by the National Center for Health Statistics (NCHS) Research Ethics Review Board. All participants signed informed consent forms.

### 2.2 Diagnostic criteria and grading standard

The inclusion criteria included: (1) alcohol consumption exceeding 28 g/day for females and 42 g/day for males over the past 12 months, (2) elevated serum aspartate aminotransferase (AST) or alanine aminotransferase (ALT) levels, with AST >25 U/L (0.42 μkat/L) for females and >35 U/L (0.58 μkat/L) for males, and (3) Total bilirubin was within normal limits <3 mg/dL (51.3 μmol/L) to exclude other conditions affecting bilirubin metabolism. The exclusion criteria included: (1) participants with hepatitis C or B infections and (2) those meeting the National Cholesterol Education Program Adult Treatment Panel III (NCEP ATP III) criteria for metabolic syndrome were excluded due to their association with elevated NAFLD risk (14). The steatosis levels S1, S2, and S3 were determined according to the median Controlled Attenuation Parameter (CAP) values ≥274 dB/m, ≥290 dB/m, and ≥302 dB/m, respectively (17).

### 2.3 Study participants and design

**Figure 1:**
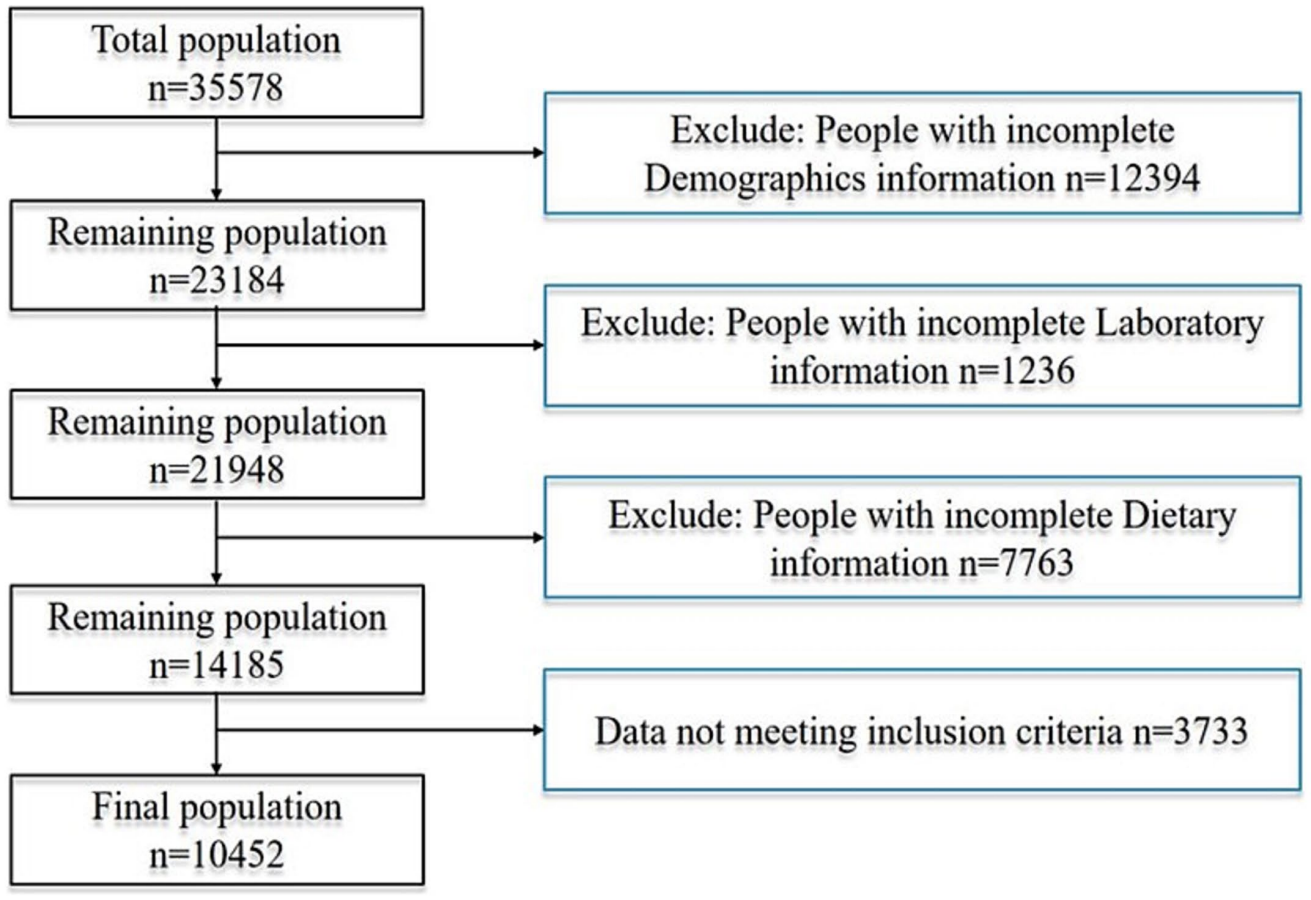
Flowchart of patient screening.

In the provided dataset, 35,578 participants, no less than 20 years old, were involved in the survey. Among them, 12,394 were excluded due to incomplete demographic information, 1,236 because of missing laboratory data, 7,763 due to a lack of complete dietary records, and 3,733 for not meeting the inclusion criteria. Consequently, 10,452 participants were included in the study, of whom 259 met the criteria for alcoholic fatty liver disease. The detailed process for inclusion and exclusion is shown in Figure 1. We applied the Benjamini–Hochberg false discovery rate (FDR) correction to control for type I error. *Post-hoc* power calculation indicated >80% power to detect an odds ratio of 0.70 for the association between serum folate and AFLD at a two-sided *α* of 0.05, thereby corroborating the adequacy of the achieved sample size.

The potential covariates in this study involved age, sex, ethnicity, marital status, household income-to-poverty ratio (PIR), educational attainment, body mass index (BMI), and smoking status. The marital status was categorized as “living alone” or “cohabiting with a partner.” The educational attainment was stratified into three groups based on years of schooling: ≤9 years, 10 to 12 years, and >12 years. Ethnicity was grouped into four categories: Mexican American, Non-Hispanic Black, Non-Hispanic White, and other ethnicities. According to a U.S. government report, household income was classified into three tiers—low, medium, and high—based on the poverty-to-income ratio (PIR) thresholds of 1.3 and 3.5. The smoking status was dichotomized into “non-smoker” (lifetime cigarette consumption <100 cigarettes) and “smoker” (≥100 cigarettes). The body mass index (BMI) was calculated as weight (kg) divided by height squared (m^2^) and categorized into two groups with a cutoff value of 25 kg/m^2^.

### 2.4 Mice model and cell culture

Eighty male C57BL/6J mice were bred in an SPF-grade animal facility and allowed free access to food and water. After a one-week acclimation period, they were divided into four groups according to their body weight randomly—normal saline control (Con), ethanol model (Et), folic acid control (FA), and folic acid + ethanol (F+Et). Ethanol exposure involved gavaging with 25, 50, and 75% liquor (56% v/v) during the first 3 weeks, followed by administration of 10 mL/kg BW of 56% v/v liquor for the next 7 weeks. Folic acid (5 mg/kg BW) was administered 1 h before ethanol gavage. All animal experiments were authorized by the medical college’s Animal Care and Use Committee at Qingdao University and adhered strictly to the National Institutes of Health’s regulations for the handling and use of laboratory animals (NO. 20201030C572720210108044). L02 human hepatocytes (from the Shanghai Institute of Cell Biology) were cultured in Dulbecco’s Modified Eagle Medium (DMEM) with 10% fetal bovine serum and 1% penicillin–streptomycin at 37 °C, 5% CO₂. The cells were processed as follows: The Con group: Cells were cultured with DMEM containing 10% fetal bovine serum for 48 h; The Et group: cells were incubated with 2.5%(v/v) ethanol for 48 h; The low/high folic acid intervention group: the Et model cells were intervened with 250 nmol/L and 500 nmol/L folic acid for 48 h. The selection of ethanol and folic acid dosages was determined by CCK8 assay, and the selected doses were based on our previous research (12).

### 2.5 Serum biochemistry assay

The hepatic enzymes ALT, AST, and serum TG were quantified using an AU5400 automatic biochemical analyzer (Beckman, Los Angeles, USA). Hepatic TG and ALT, as well as AST levels, in L02 cell supernatant were assessed following the manufacturer’s guidelines (Jiancheng, Nanjing, China).

### 2.6 Histological analysis

Liver tissue samples were fixed in 10% neutral buffered formalin, embedded in paraffin, and sectioned at 4-μm thickness. Hematoxylin and eosin (H&E) staining was then performed on these sections. For L02 cells, Oil Red O staining was conducted according to the manufacturer’s instructions (Solarbio, Beijing, China). Histopathological alterations in the liver sections and cells were examined using an ABX60 light microscope (Olympus, Tokyo, Japan).

### 2.7 Western blot

Proteins were separated on 10%/6% SDS-polyacrylamide gels and transferred to PVDF membranes (Millipore, Billerica, MA, USA), which were activated with methanol. The membranes were blocked with 10% skim milk to reduce non-specific binding. They were then incubated overnight at 4 °C with primary antibodies against SREBP-1c (1:1000 dilution, ABclonal, Wuhan, China), FASN (1:1000 dilution, Abways, Shanghai, China), ACC1 (1:1000 dilution, Abcam, Cambridge, UK), SCD-1 (1:1000 dilution, Affinity, Wuhan, China), and β-actin (1:10,000 dilution, Affinity, Wuhan, China). After washing with TBST, the membranes were incubated with secondary antibodies (1:10,000) for 1 h.

### 2.8 Statistical analysis

In this study, continuous variables are presented as mean ± SD with statistical differences among each group compared by ANOVA. For categorical variables, 95% confidence intervals (95% CI) were used for statistical descriptions and chi-square tests for comparisons. Logistic regression models were applied to calculate the odds ratios (ORs) and 95% CI for AFLD in relation to folate indexes. Model 1 was unadjusted. Model 2 was adjusted for sociodemographic factors (age, sex, ethnicity, education, marital status). Model 3 further adjusted for BMI, waist circumference, smoking, and poverty–income ratio (PIR) based on Model 2. Restricted cubic spline (RCS) analysis was performed to explore the non-linear relationship between folate and AFLD risk. Subgroup analyses were conducted to explore the association between the folate indicators and AFLD in different subgroups, based on age, gender, smoking behavior, BMI, education level, marital status, and family PIR. To minimize potential biases inherent in the NHANES database, we applied strict inclusion and exclusion criteria, excluded participants with incomplete demographic, biochemical, or dietary data, and adjusted for a comprehensive set of covariates (age, sex, ethnicity, education, marital status, BMI, smoking status, and PIR) in multivariable regression models. Sensitivity analyses were conducted to evaluate the robustness of the findings. R software (4.2.1) and GraphPad Prism 8.0 (GraphPad, San Diego, CA, USA) were used for statistical analysis, with a *p*-value of <0.05 as statistically significant.

## 3 Results

### 3.1 Baseline characteristics of participants

Among the included participants (Table 1), the mean age was 44.3 ± 0.36 years. The mean age of individuals diagnosed with alcoholic fatty liver disease (AFLD) was 44.57 ± 1.08 years, with males accounting for 52.97% of this group, and the majority being non-Hispanic white (80.25%). Of the AFLD patients, 68.08% had an educational level above high school, 71.78% were married or living with a partner, and the mean household income-to-poverty ratio (PIR) was 3.42. The daily alcohol consumption of AFLD patients was 67.73 ± 3.94 g, which was significantly higher than that of non-AFLD patients (*p* < 0.01). Additionally, serum AST and ALT levels were significantly higher in AFLD patients compared to non-AFLD patients (both *p* < 0.01). A greater proportion of AFLD patients had low serum folate levels, whereas the opposite trend was observed for red blood cell folate, with a higher proportion of AFLD patients exhibiting high levels.

**Table 1:** Baseline characteristics of participants in NHANES 2011–2020.

Variable	Total (*n* = 10,452)	No AFLD (*n* = 10,193)	AFLD (*n* = 259)	*p*-value
Age (year)	44.30 (0.36)	44.29 (0.36)	44.57 (1.08)	0.79
Gender (%)				0.06
Male	4,874 (45.70)	4,732 (45.47)	142 (52.97)	
Female	5,578 (54.30)	5,461 (54.53)	117 (47.03)	
Ethnicity (%)				**<0.01**
Non-Hispanic White	4,051 (67.12)	3,921 (66.69)	130 (80.25)	
Non-Hispanic Black	2,475 (10.98)	2,413 (11.06)	62 (8.39)	
Mexican American	1,198 (7.57)	1,177 (7.70)	21 (3.75)	
Other Hispanic	1,005 (6.07)	980 (6.14)	25 (3.92)	
Other ethnicities	1723 (8.26)	1702 (8.41)	21 (3.69)	
Education level (%)				0.24
Less than high school	1,659 (10.77)	1,621 (10.88)	38 (7.51)	
High school	2,197 (20.42)	2,129 (20.29)	68 (24.41)	
More than high school	6,596 (68.80)	6,443 (68.83)	153 (68.08)	
Marital status				0.06
Married/living with partner	6,152 (63.25)	5,986 (62.97)	166 (71.78)	
Widowed/divorced/separated	1880 (15.51)	1836 (15.61)	44 (12.37)	
Never married	2,420 (21.24)	2,371 (21.42)	49 (15.86)	
Family PIR	3.11 (0.05)	3.10 (0.05)	3.42 (0.15)	**0.02**
BMI (kg/m^2^)	27.35 (0.10)	27.37 (0.10)	26.97 (0.53)	0.45
Waist (cm)	94.48 (0.23)	94.48 (0.22)	94.34 (1.39)	0.92
Alcohol consumption (g)	10.77 (0.41)	8.89 (0.34)	67.73 (3.94)	**<0.01**
Smoking behavior (%)				**<0.01**
Never	6,331 (60.50)	6,229 (61.18)	102 (39.77)	
Former	2,194 (22.48)	2,137 (22.42)	57 (24.25)	
Now	1927 (17.02)	1827 (16.40)	100 (35.98)	
AST (IU/L)	23.93 (0.17)	23.17 (0.17)	46.99 (2.87)	**<0.01**
ALT (IU/L)	22.77 (0.21)	21.96 (0.18)	47.34 (3.37)	**<0.01**
Total bilirubin μmol/L	10.38 (0.13)	10.35 (0.13)	11.23 (0.45)	0.05
Platelet count 1,000 cells/μL	238.10 (1.06)	238.42 (1.08)	228.20 (5.18)	0.06
DFE (mcg)				0.12
Q1 (<286 mcg)	2,604 (22.77)	2,560 (23.00)	44 (15.86)	
Q2 (286 ~ 403.8 mcg)	2,614 (25.67)	2,537 (25.43)	77 (32.97)	
Q3 (403.8 ~ 585 mcg)	2,621 (25.21)	2,558 (25.19)	63 (25.79)	
Q4 (≥585 mcg)	2,613 (26.35)	2,538 (26.38)	75 (25.37)	
Serum folate (nmol/L)				**< 0.01**
Q1 (<0.449 nmol/L)	2,579 (22.62)	2,478 (22.10)	101 (38.31)	
Q2 (0.449 ~ 0.595 nmol/L)	2,613 (25.26)	2,558 (25.40)	55 (21.06)	
Q3 (0.595 ~ 0.842 nmol/L)	2,645 (26.12)	2,584 (26.19)	61 (23.79)	
Q4 (≥0.842 nmol/L)	2,615 (26.00)	2,573 (26.30)	42 (16.84)	
5-MTHF (nmol/L)				0.18
Q1 (<21.3 nmol/L)	2,612 (21.95)	2,521 (21.77)	91 (27.47)	
Q2 (21.3 ~ 31.4 nmol/L)	2,602 (23.24)	2,540 (23.17)	62 (25.29)	
Q3 (31.4 ~ 47 nmol/L)	2,623 (26.64)	2,573 (26.83)	50 (20.81)	
Q4 (≥47 nmol/L)	2,615 (28.17)	2,559 (28.23)	56 (26.44)	
RBC folate (nmol/L)				**0.03**
Q1 (<789.3 nmol/L)	2,610 (20.59)	2,547 (20.59)	63 (20.64)	
Q2 (789.3 ~ 1,020 nmol/L)	2,545 (23.71)	2,489 (24.05)	56 (13.43)	
Q3 (1,020 ~ 1,320 nmol/L)	2,670 (27.31)	2,608 (27.17)	62 (31.51)	
Q4 (≥1,320 nmol/L)	2,627 (28.39)	2,549 (28.19)	78 (34.42)	

Bold values indicate statistical significance.

### 3.2 The association between folate-related indicators and AFLD

**Figure 2:**
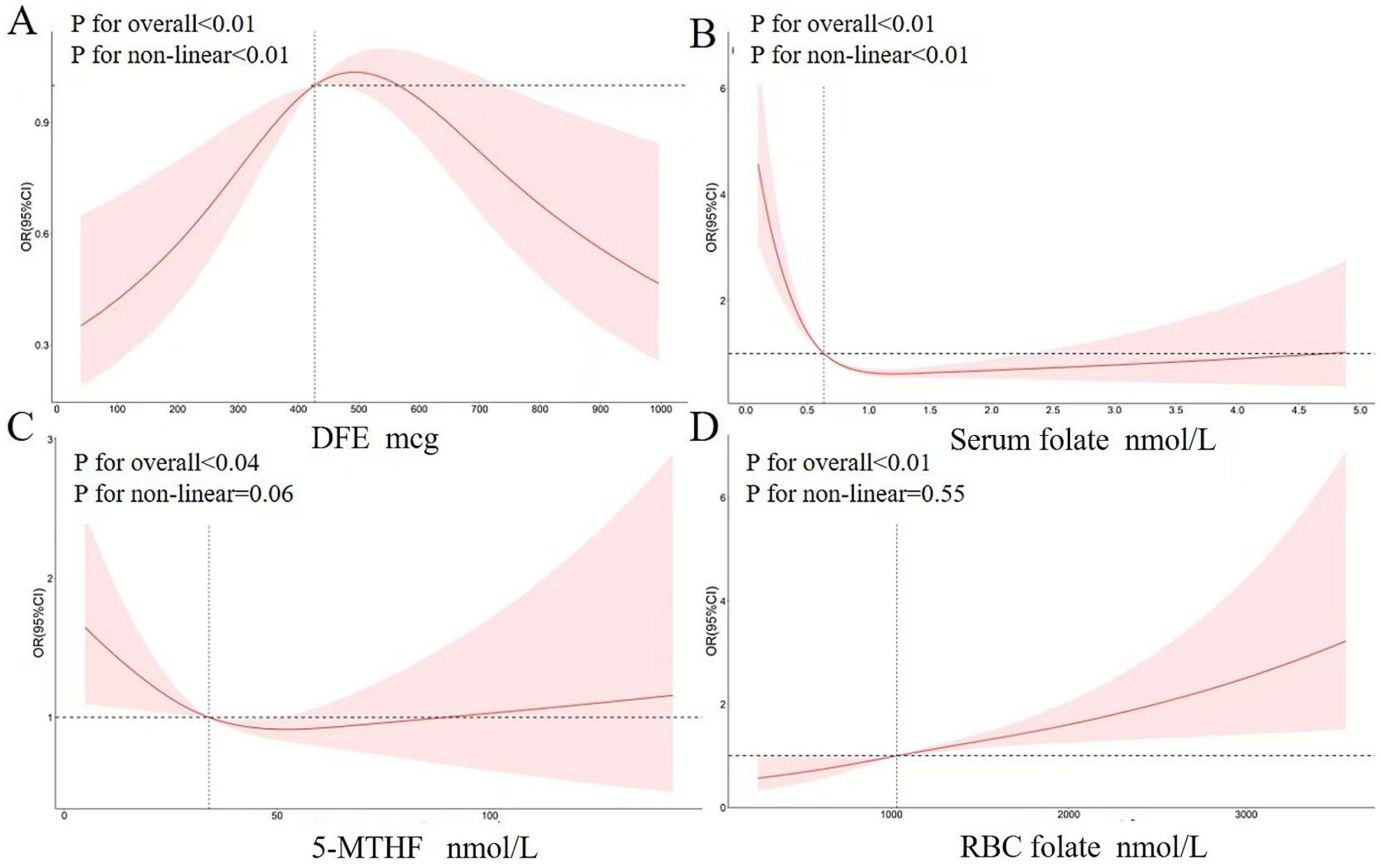
Restricted cubic spline plot of folate-related indicators **(A)** DFE **(B)** Serum folate **(C)** 5-MTHF **(D)** RBC folate and AFLD. The solid and pink lines indicate predictions and 95% confidence intervals, which were adjusted for age, sex, ethnicity, education, marital status, BMI, waist circumference, smoking, and poverty–income ratio.

Further analysis of the relationship between AFLD and folate was conducted using three multiple logistic regression models (Table 2). In the unadjusted Model 1, compared to the lowest serum folate quartile, higher serum folate levels showed a significant negative correlation with AFLD (OR2 = 0.48, OR3 = 0.52, OR4 = 0.37). For other folate-related indicators, compared to the lowest 5-MTHF quartile, OR3 for Q3 was 0.61. Compared to the lowest red blood cell folate quartile, OR2 for Q2 was 0.56. In Model 2, after adjusting for age, sex, ethnicity, education, and marital status, serum folate still showed a significant negative correlation with AFLD (OR2 = 0.44, OR3 = 0.49, OR4 = 0.33). For other folate-related indicators, compared to the lowest 5-MTHF quartile, OR3 for Q3 was 0.59. Compared to the lowest red blood cell folate quartile, OR2 for Q2 was 0.54. Model 3 further adjusted for BMI, waist circumference, smoking, and poverty–income ratio based on Model 2. The results still showed a significant negative correlation between serum folate and AFLD (OR2 = 0.45, OR3 = 0.50, OR4 = 0.35). RCS analysis of folate-related indicators and AFLD is shown in Figure 2. When considering all confounding covariates, serum folate levels (0.636 ~ 5.0 nmol/L) showed a non-linear correlation with AFLD, which reinforced the concept that serum folate acts as a protective factor in ethanol-induced hepatic steatosis.

**Table 2:** Association between DFE, serum folate, 5-MTHF, and RBC folate with AFLD participants in NHANES 2011–2020.

Variable	Model 1		Model 2		Model 3	
	OR (95%CI)	*p-*value	OR (95%CI)	*p-*value	OR (95%CI)	*p-*value
DFE (mcg)						
Q1 (<286 mcg)	REF					
Q2 (286 ~ 403.8 mcg)	1.88 (1.15,3.06)	**0.01**	1.78 (1.09,2.91)	**0.02**	1.93 (1.16,3.19)	**0.01**
Q3 (403.8 ~ 585 mcg)	1.48 (0.88,2.49)	0.13	1.40 (0.82,2.39)	0.21	1.55 (0.89,2.70)	0.12
Q4 (>585 mcg)	1.39 (0.83,2.35)	0.21	1.26 (0.74,2.16)	0.38	1.43 (0.83,2.46)	0.20
Serum folate (nmol/L)						
Q1 (<0.449 nmol/L)	REF					
Q2 (0.449 ~ 0.595 nmol/L)	0.48 (0.32,0.73)	**<0.01**	0.44 (0.29,0.68)	**<0.01**	0.45 (0.29,0.71)	**<0.01**
Q3 (0.595 ~ 0.842 nmol/L)	0.52 (0.31,0.88)	**0.02**	0.49 (0.28,0.83)	**0.01**	0.50 (0.29,0.85)	**0.01**
Q4 (≥0.842 nmol/L)	0.37 (0.24,0.57)	**<0.01**	0.33 (0.21,0.52)	**<0.01**	0.35 (0.22,0.54)	**<0.01**
5-MTHF (nmol/L)						
Q1 (<21.3 nmol/L)	REF					
Q2 (21.3 ~ 31.4 nmol/L)	0.87 (0.56,1.33)	0.50	0.88 (0.57,1.36)	0.56	0.89 (0.57,1.41)	0.62
Q3 (31.4 ~ 47 nmol/L)	0.61 (0.40,0.95)	**0.03**	0.59 (0.37,0.93)	**0.02**	0.63 (0.40,1.00)	0.052
Q4 (≥47 nmol/L)	0.74 (0.47,1.16)	0.19	0.69 (0.43,1.12)	0.13	0.78 (0.47,1.32)	0.35
RBC folate (nmol/L)						
Q1 (<789.3 nmol/L)	REF					
Q2 (789.3 ~ 1,020 nmol/L)	0.56 (0.33,0.93)	**0.03**	0.54 (0.31,0.91)	**0.02**	0.59 (0.34,1.03)	0.06
Q3 (1,020 ~ 1,320 nmol/L)	1.16 (0.69,1.93)	0.57	1.07 (0.63,1.81)	0.81	1.22 (0.69,2.14)	0.49
Q4 (≥1,320 nmol/L)	1.22 (0.82,1.82)	0.33	1.11 (0.70,1.75)	0.66	1.33 (0.81,2.20)	0.26

Bold values indicate statistical significance.

### 3.3 The relationship between folate-related indicators and advanced steatosis

After adjusting for confounding factors and analyzing the relationship between folate levels and hepatic steatosis, it was found that there are significant differences in serum 5-MTHF and RBC folate levels between patients with AFLD and non-AFLD patients (Table 3). Serum 5-MTHF levels showed a downward trend with the progression of steatosis (*p* for trend <0.01). Additionally, patients with S3 steatosis had lower dietary folate levels than non-AFLD patients, while those with S2 steatosis had lower serum folate levels, and these differences were statistically significant.

**Table 3:** Association of DFE, serum folate, 5-MTHF, and RBC folate with alcoholic liver steatosis in participants from NHANES 2011–2020.

Variable	Overall difference			Categories of liver steatosis								
	Overall	*p*-value (liver steatosis vs. non-liver steatosis)	Non-AFLD	AFLD	S1	*p*-value (vs. non-liver steatosis)	S2	*p*-value (vs. non-liver steatosis)	S3	*p*-value (vs. non-liver steatosis)	*p*-value (S1 vs. S2 vs. S3)	*p* for trend
DFE (mcg)	483.73 (9.34)	0.14	490.52 (11.61)	467.36 (11.97)	489.99 (17.02)	0.98	466.58 (26.18)	0.38	455.39 (12.50)*	**0.04**	0.22	0.1
Serum folate (nmol/L)	1.46 (0.16)	0.16	1.55 (0.19)	1.25 (0.16)	1.59 (0.32)	0.89	0.91 (0.15)*	**0.01**	1.18 (0.21)	0.18	0.09	0.19
5-MTHF (nmol/L)	38.07 (0.82)	0.01	38.61 (0.86)	36.78 (0.92)	41.60 (1.94)	0.10	34.71 (1.70)	0.38	34.82 (1.09)**	**<0.01**	**0.01**	**<0.01**
RBC folate (nmol/L)	1149.42 (14.76)	<0.01	1126.43 (14.89)	1204.79 (20.02)	1234.73 (31.38)**	**<0.01**	1136.42 (33.23)	0.77	1209.47 (32.80)*	**0.01**	**0.05**	0.41

*p < 0.05 and **p < 0.01 compared to non-AFLD. Bold values indicate statistical significance.

### 3.4 Subgroup analysis

Subgroup analysis showed that RBC folate was a significant risk factor in multiple subgroups, including people aged 65 and older (OR = 6.04), males (OR = 2.15), current smokers (OR = 2.42), those with education below high school (OR = 2.26), married/cohabiting individuals (OR = 2.07), and middle/high-income groups (OR = 2.23 ~ 2.25). 5-MTHF had a protective effect on females (OR = 0.61) and low-income populations (OR = 0.56). Serum folate was associated with reduced risk in high-income groups (OR = 0.16). In summary, RBC folate is a core risk indicator across subgroups, while 5-MTHF and serum folate have potential protective effects in specific populations (Table 4).

**Table 4:** Subgroup analysis of DEF, serum folate, 5-MTHF, and RBC folate in AFLD participants in NHANES 2011–2020.

Characteristic	DFE		Serum folate		5-MTHF		RBC folate	
	OR (95%CI)	*p-*value	OR (95%CI)	*p-*value	OR (95%CI)	*p-*value	OR (95%CI)	*p-*value
Age (year)								
<65	1.16 (0.88, 1.54)	0.28	0.82 (0.55, 1.23)	0.33	0.74 (0.53, 1.03)	0.08	**1.75 (1.06, 2.88)**	**0.03**
≥65	1.53 (0.36, 6.51)	0.56	1.13 (0.69, 1.83)	0.63	2.28 (0.83, 6.22)	0.11	**6.04 (1.47, 24.72)**	**0.01**
Gender								
Male	1.27 (0.84, 1.92)	0.25	0.66 (0.36, 1.23)	0.19	1.02 (0.62, 1.65)	0.95	**2.15 (1.06, 4.35)**	**0.03**
Female	1.16 (0.79, 1.69)	0.45	1.00 (0.70, 1.42)	0.99	**0.61 (0.41, 0.91)**	**0.02**	1.86 (0.97, 3.60)	0.06
Smoking behavior								
Never	1.33 (0.88, 2.02)	0.17	0.81 (0.48, 1.39)	0.44	0.66 (0.41, 1.07)	0.09	1.68 (0.77, 3.64)	0.19
Former	0.84 (0.40, 1.76)	0.63	0.75 (0.35, 1.61)	0.45	0.76 (0.40, 1.44)	0.39	2.16 (0.69, 6.75)	0.18
Now	1.28 (0.86, 1.91)	0.22	0.99 (0.56, 1.77)	0.98	1.15 (0.64, 2.05)	0.64	**2.42 (1.15, 5.13)**	**0.02**
BMI (kg/m^2^)								
<25	0.98 (0.58, 1.64)	0.93	1.00 (0.67, 1.48)	0.98	0.91 (0.55, 1.51)	0.70	1.86 (0.96, 3.62)	0.07
≥25	1.39 (0.95, 2.05)	0.09	0.67 (0.40, 1.11)	0.12	0.72 (0.48, 1.08)	0.11	1.93 (0.98, 3.76)	0.06
Education level								
Less than high school	1.82 (1.01, 3.28)	0.05	1.01 (0.73, 1.38)	0.97	0.79 (0.55, 1.12)	0.18	**2.26 (1.35, 3.77)**	**<0.01**
High school	1.24 (0.76, 2.04)	0.39	0.49 (0.24, 0.99)	0.05	0.85 (0.41, 1.76)	0.66	1.42 (0.59, 3.40)	0.43
More than high school	1.06 (0.75, 1.50)	0.74	**0.16 (0.06, 0.41)**	**<0.01**	0.83 (0.41, 1.68)	0.60	1.97 (0.49, 7.96)	0.34
Marital status								
Married/living with partner	1.00 (0.69, 1.45)	0.99	0.92 (0.62, 1.38)	0.69	0.91 (0.63, 1.34)	0.64	**2.07 (1.20, 3.58)**	**0.01**
Widowed/divorced/separated	2.23 (1.19, 4.16)	**0.01**	0.76 (0.37, 1.56)	0.45	0.62 (0.27, 1.42)	0.25	2.68 (0.92, 7.81)	0.07
Never married	1.38 (0.89, 2.13)	0.15	0.56 (0.32, 0.99)	0.05	0.58 (0.27, 1.25)	0.16	1.04 (0.38, 2.83)	0.94
Family PIR								
≤1.30	1.70 (1.11, 2.61)	**0.02**	0.61 (0.31, 1.21)	0.15	**0.56 (0.33, 0.95)**	**0.03**	1.27 (0.65, 2.47)	0.48
1.30–3.50	1.16 (0.71, 1.89)	0.54	0.76 (0.39, 1.50)	0.42	0.97 (0.50, 1.89)	0.94	**2.23 (1.13, 4.40)**	**0.02**
>3.50	1.03 (0.65, 1.64)	0.88	0.95 (0.63, 1.43)	0.79	0.86 (0.56, 1.32)	0.49	**2.25 (1.11, 4.55)**	**0.02**

Bold values indicate statistical significance.

### 3.5 Intervention effects of folic acid on AFLD

**Figure 3:**
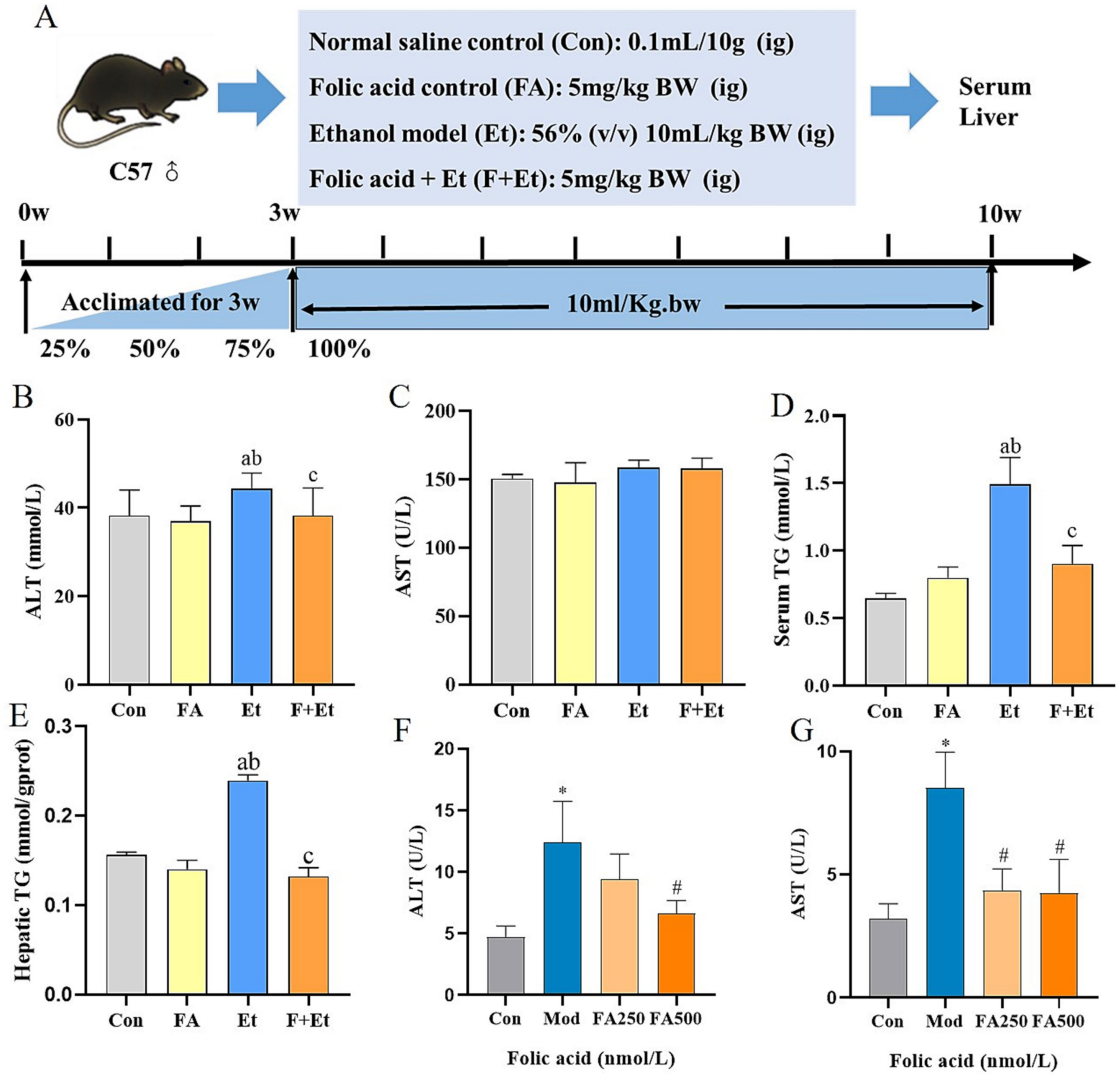
Folic acid administration improves ethanol-induced liver enzymes and hepatic TG levels. **(A)** Study design showing groups and durations of ethanol and folic acid treatments. **(B)** Serum ALT and **(C)** AST levels, **(D)** Serum TC, **(E)** hepatic TG levels, **(F)** ALT levels, and **(G)** AST levels measured in culture medium, values are expressed as mean ± SEM (*n* = 3). *p < 0.05, significantly different compared to the control group, ^#^*p* < 0.05, significantly different compared to the ethanol-treated model group.

During the experimental period, no statistically significant differences were observed in body weight among the groups (Figure 3). The liver index was elevated in ethanol-exposed mice, showing a significant difference compared to the Con group (*p* < 0.05). After folic acid intervention, the liver index remained different from the Con group (*p* < 0.05), but no significant difference was observed compared to the FA group (*p* > 0.05) (detailed results are provided in Supplementary materials).

Ethanol exposure significantly increased hepatic ALT levels by 15.9 and 18.4% compared to the Con and FA groups, respectively (*p* < 0.05). Folic acid intervention notably reduced ALT levels, restoring them to near-normal ranges. Although serum AST levels in the ethanol-exposed group showed an upward trend, the difference was not statistically significant (*p* > 0.05). Additionally, ethanol exposure for 48 h increased ALT and AST levels in the supernatant of L02 cells by 2.62-fold and 2.64-fold, respectively. Folic acid intervention reduced these levels, with the high-dose folic acid group showing a significant reduction in ethanol-induced ALT and AST levels compared to the model group (*p* < 0.05).

Serum lipid profile analysis revealed that ethanol exposure increased serum and hepatic TG levels by 130.0 and 56.1%, respectively, compared to the Con group (*p* < 0.05). Folic acid intervention reduced these levels by 39.4 and 43.5%, respectively, showing statistically significant differences compared to the Et group (*p* < 0.05).

### 3.6 Pathological alterations of AFLD ameliorated by folic acid

**Figure 4:**
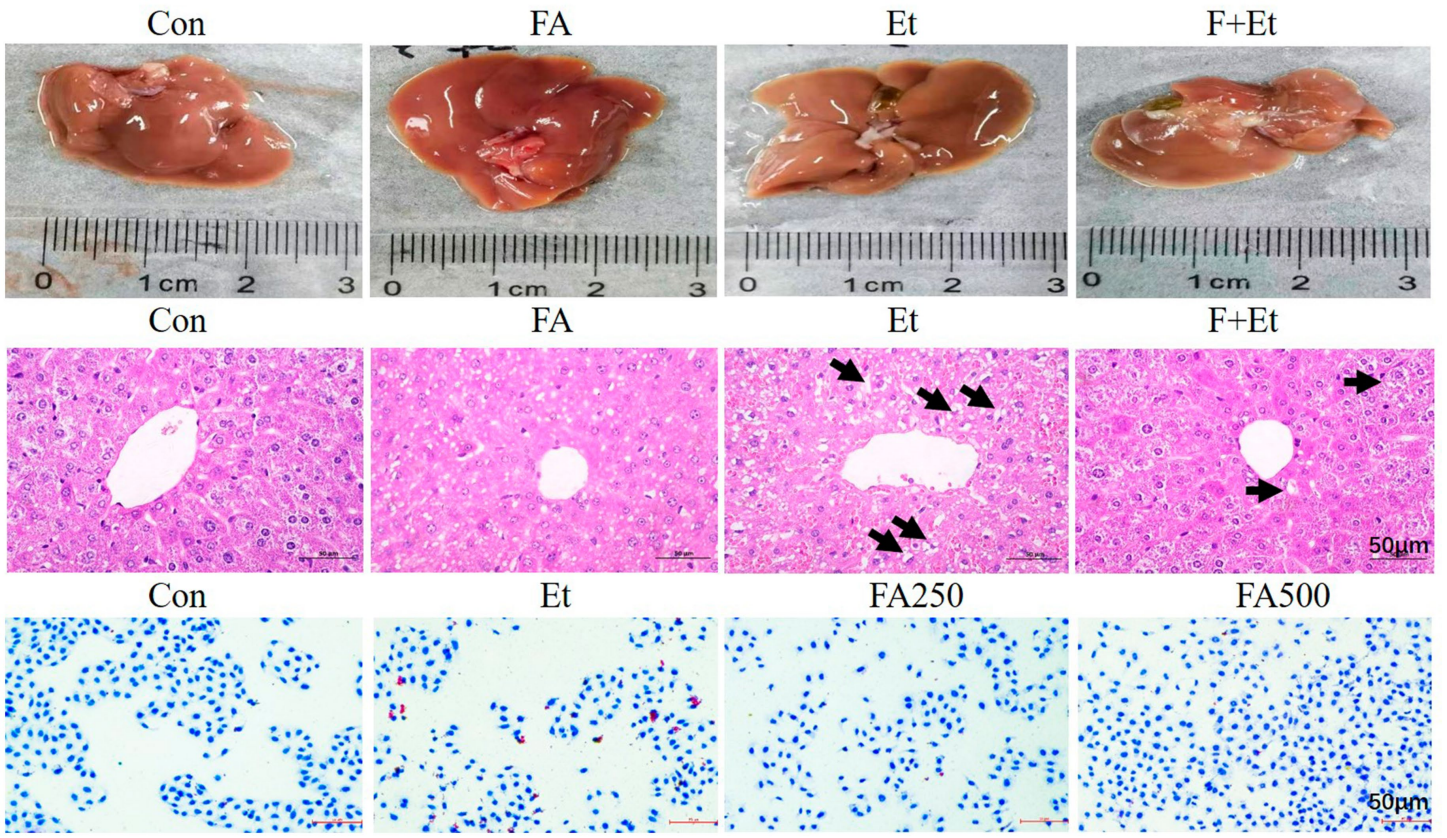
Representative images of liver appearance, liver sections by H&E staining of liver sections, and oil red staining in L02 cells (×50, bar = 200 μm). The lipid droplet was denoted with blue arrows in the H&E image.

Macroscopically, livers from the Con and FA control groups exhibited a dark red color with uniform texture and regular morphology. In contrast, ethanol-exposed livers displayed hemorrhagic spots and a yellowish tint, which normalized after folic acid intervention. Histopathological examination revealed intact hepatic cords radiating from the central vein in the Con and FA groups. The liver tissues of the Et group exhibited significant pathological damage, including hepatocyte congestion and swelling, disorganized hepatic cord arrangement, enlarged hepatocytes containing single large circular lipid droplets (indicated by black arrows), and nuclear displacement. Oil Red O staining demonstrated abundant lipid droplets distributed in a ring-like pattern along the inner cell membrane in L02 cells after 48 h of ethanol exposure. Low-dose folic acid intervention slightly reduced lipid droplets, while high-dose folic acid significantly decreased their accumulation (Figure 4).

### 3.7 Effects of folic acid on key proteins in AFLD lipid metabolism

**Figure 5:**
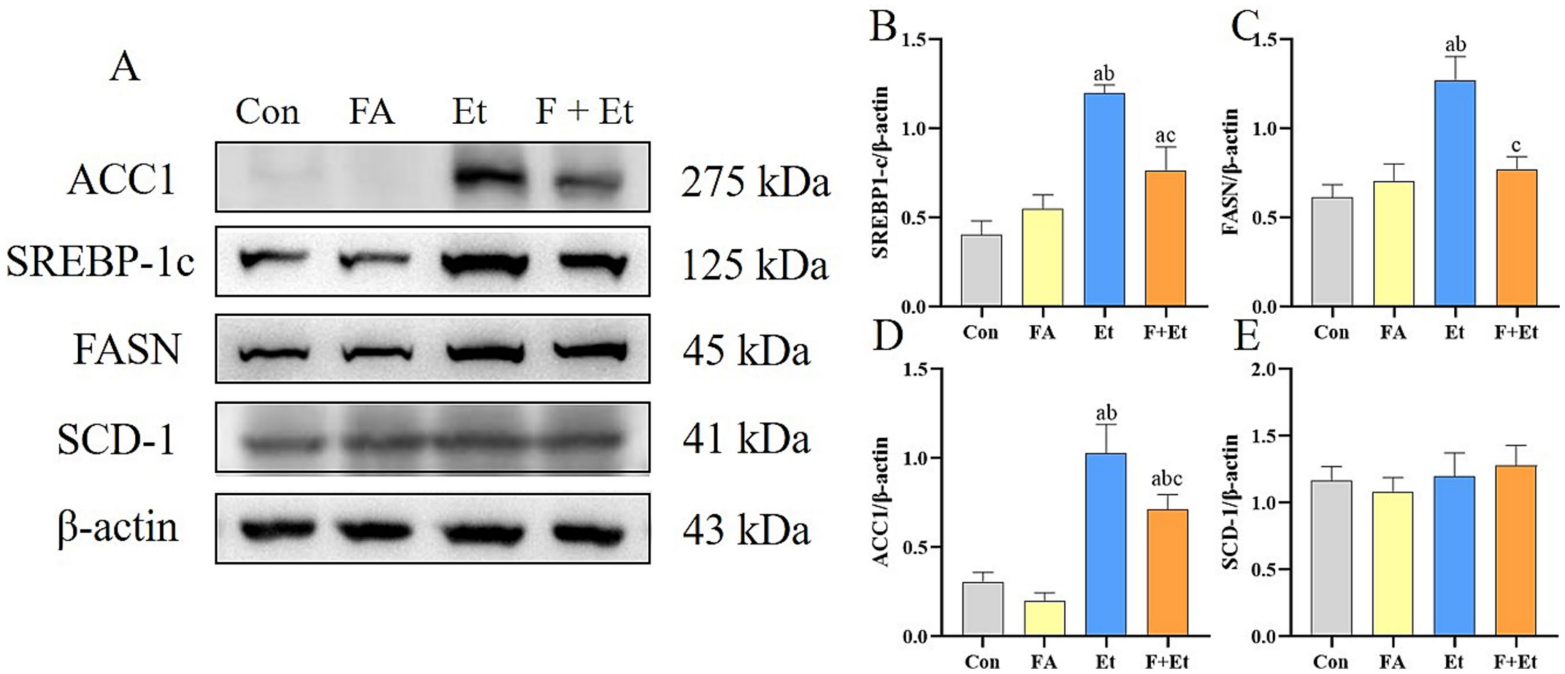
Effects of folic acid on the protein expression of TG synthesis. Values are expressed as mean ± SEM (*n* = 3). **(A)** Hepatic protein bands and relative levels of the SREBP1-c **(B)**, FASN **(C)**, ACC **(D)**, and SCD-1 **(E)** proteins. A significant difference (*p* < 0.05) is identified by different letters: a, vs. the Con group; b, vs. the FA control group; and c, vs. the Et group.

The expression levels of TG synthesis-related proteins are shown in Figure 5. In the Et groups, the expression levels of SREBP-1c, FASN, and ACC1 increased by 2.96-, 2.07-, and 3.34-fold, respectively, compared to the Con groups. Folic acid intervention markedly inhibited the expression of these proteins (*p* < 0.05). However, no obvious change in SCD-1 expression was observed in the F+Et group compared to the Et group (*p* > 0.05).

## 4 Discussion

Nearly 90–100% of chronic heavy drinkers develop alcohol-induced hepatic steatosis (where fat exceeds 5% of liver weight). Consequently, ethanol is often categorized as a pure energy substrate, providing 7 kcal/g (18). Conventionally, alcohol-induced hepatic steatosis is attributed to ethanol metabolism producing acetate, which fuels *de novo* lipogenesis. However, the liver primarily recognizes ethanol as a toxin rather than as a nutritive energy source. Thus, the caloric energy from ethanol is not stored but is rapidly dissipated (19). This metabolic process involves substantial consumption of mitochondrial nicotinamide adenine dinucleotide (NAD+) and generates abundant reactive oxygen species (ROS), culminating in the production of water and carbon dioxide. Only a minor fraction is converted into macromolecules, such as proteins or fatty acids, for utilization (20). Consequently, the liver reduces its reliance on conventional energy substrates, such as fatty acids, leading to their accumulation within hepatocytes and resulting in steatosis.

Ethanol metabolism generates excessive ROS and places a high metabolic burden on mitochondria, impairing mitochondrial function and inducing mitophagy overactivation (21). This leads to the formation of megamitochondria and is associated with elevated intrinsic apoptotic signaling, manifesting as abnormal liver enzyme levels (22). Accumulating evidence suggests that folic acid deficiency and impaired folic acid metabolism exacerbate the progression of hepatic steatosis, particularly since folic acid storage and metabolism predominantly occur in the liver (23). Approximately 90% of alcohol is metabolized hepatically, causing injury through mechanisms including oxidative stress, gut-derived inflammatory mediators, endotoxemia, and nutritional imbalances (19). Therefore, we propose a significant relationship between folic acid status and alcohol-induced liver injury. The data from the NHANES survey indicate that serum folate and 5-MTHF levels act as protective factors against AFLD in a specific range. Notably, 5-MTHF levels exhibit an inverse correlation with the degree of hepatic steatosis. Published research reported that epigenetic alterations, particularly DNA methylation, play a crucial role in insulin resistance (24, 25). A cross-sectional study by Li et al. involving 1,530 non-diabetic US adults found an inverse association between serum folate and insulin resistance: A 25% increase in serum folate correlated with a 3.06% decrease in HOMA-IR and a 2.77% decrease in insulin levels (26). Lopez et al. demonstrated that folic acid deficiency is linked to hypomethylation of the CAMKK2 gene, which regulates glucose metabolism and directly influences HOMA-IR levels (27). Our previous research also found that folic acid intervention attenuates ethanol-induced increases in DNMT3a levels, thereby modulating hepatic DNA methylation patterns (28). This suggests that folic acid’s regulatory effect on hepatic steatosis may involve alterations in DNA methylation.

Paradoxically, our results indicate that AFLD patients exhibit higher red blood cell folate levels compared to non-AFLD individuals (Table 3). Subgroup analyses further suggest that in specific individuals, elevated RBC folate may be a risk factor for AFLD. Unlike serum folate levels, which reflect recent intake, absorption, and storage, RBC folate represents long-term folate status and body stores. Li et al. (26) identified RBC folate as an independent risk factor for increased NAFLD risk and its association with metabolic diseases such as diabetes. Studies in morbidly obese individuals also show strong correlations between RBC folate levels, insulin resistance, and metabolic syndrome (29). Notably, heavy drinkers exhibit a higher proportion of abnormally shaped erythrocytes, including characteristic triangular forms and elevated mean corpuscular volume (MCV), which correlates with the amount and duration of alcohol intake. This hematological abnormality may stem from impaired hematopoiesis due to acetaldehyde metabolism within the bone marrow hematopoietic stem cells (30). Folate is primarily incorporated into developing erythrocytes during the early stages (reticulocyte stage) in the bone marrow. Once mature erythrocytes enter circulation, the folate content becomes relatively stable and neither metabolizes nor actively takes up new folate (31). Beyond these morphological and functional alterations, chronic alcohol intake also disrupts vitamin B12 metabolism. Although serum B12 levels in AFLD patients are not necessarily reduced and may even appear elevated due to hepatic release during liver injury, hepatic utilization of vitamin B12 is impaired, resulting in a state of functional deficiency (32). This deficiency blocks the methionine synthase-dependent remethylation pathway, creating a “methyl-folate trap” that prevents effective folate utilization (33). During early erythropoiesis, when DNA/RNA synthesis and hemoglobin production rely on active one-carbon metabolism, such impairment may result in folate retention within developing erythrocytes, manifesting as elevated RBC folate (34). Thus, both impaired folate–vitamin B12 metabolism and alcohol-induced abnormalities in erythropoiesis may jointly explain the paradoxical increase in RBC folate observed in AFLD patients. The precise mechanisms still require further elucidation.

Based on the epidemiological correlation between folate and alcoholic hepatic steatosis identified in a cross-sectional survey, the causal relationship was further explored *in vivo/in vitro* intervention studies. Our results demonstrate that folic acid supplementation inhibits alcohol-induced elevations in serum ALT, serum TG, and hepatic TG, while reducing the number of cytoplasmic lipid droplets, which indicates a protective effect on alcohol consumption. Mechanistically, folic acid significantly downregulates key hepatic lipogenic enzymes—the transcriptional activator SREBP-1c, acetyl-CoA carboxylase (ACC1), catalyzing the rate-limiting step of malonyl CoA synthesis, and fatty acid synthase (FASN), driving palmitate production, but does not affect stearoyl-CoA desaturase 1 (SCD-1). However, the precise molecular mechanism remains incompletely understood. One plausible hypothesis is that folic acid modulates DNMT3a activity or recruitment, thereby influencing SREBP-1c-driven transcriptional programs. DNMT3a is a *de novo* DNA methyltransferase that has been implicated in the epigenetic regulation of lipid metabolism. For example, it has been reported that intestinal FGF15/19 recruits DNMT3a to the FASN promoter, resulting in methylation-dependent transcriptional repression and subsequent suppression of de novo lipogenesis (35). These findings raise the possibility that folic acid, by fueling one-carbon metabolism and supporting DNMT3a-mediated methylation, could indirectly dampen SREBP-1c signaling through repression of downstream lipogenic genes. Alternatively, folic acid may act through stress-related mechanisms. Oxidative stress, mitochondrial dysfunction, and endoplasmic reticulum (ER) stress are well-established upstream activators of SREBP-1c. Indeed, mitochondrial stress has been shown to promote hepatic lipid accumulation via the HSP60-mTORC1-SREBP-1c axis (36), while ER stress itself can trigger SREBP-1c activation and aberrant lipid and glucose metabolism. Given that folic acid supplementation alleviates oxidative and mitochondrial stress in alcoholic liver disease (12), its inhibitory effect on SREBP-1c may, at least in part, be mediated by the mitigation of these stress responses. The lack of effect on SCD-1 suggests that folic acid preferentially regulates *de novo* lipogenesis rather than fatty acid desaturation. Given that SCD-1 acts downstream to convert saturated fatty acids into monounsaturated fatty acids (37), its unchanged expression may reflect a selective mechanism whereby folic acid suppresses lipid synthesis while preserving desaturation pathways essential for cellular homeostasis. Further studies are warranted to confirm this specificity.

AFLD is characterized by excessive hepatic triglyceride accumulation, which is driven primarily by mitochondrial dysfunction and endoplasmic reticulum (ER) stress rather than direct incorporation of ethanol-derived acetate (38). Our previous studies indicate that folic acid intervention significantly attenuates ethanol-induced swelling, vesiculation of ER, and the PERK-eIF2α, p-IRE1-XBP-1, and ATF6 pathways activation, which both orchestrate various aspects of fatty acid and lipid metabolism independently and in an integrated manner (13). Furthermore, alcohol exposure and folate deficiency are both implicated in inducing mitochondrial dysfunction and reducing mitochondrial quantity, thereby impairing cellular energy metabolism. In undifferentiated cells, folate specifically targets and activates mitochondrial function. It upregulates the PGC-1α-mediated mitochondrial biogenesis pathway while concurrently downregulating the expression of the mitochondrial fission protein Drp1 (39). This dual action promotes mitochondrial elongation, enhances ATP generation capacity, and mitigates intracellular oxidative damage. Structurally, folic acid contains a pteridine ring with a hydroxyl group capable of scavenging free radicals, conferring antioxidative properties, and reducing ROS-mediated damage (40). In addition, folate improves one-carbon metabolism by lowering homocysteine (Hcy) levels, thereby alleviating redox imbalance and supporting methylation reactions required for hepatocellular function. Building upon our group’s prior research, folic acid supplementation ameliorates elevated serum homocysteine (Hcy) levels and alleviates excessive mitophagy, which restores mitochondrial functionality in ALD mice (12).

Mitochondria and the ER function as distinct organelles; however, they interact dynamically through mitochondria-associated ER membranes (MAMs) (41). These structures critically regulate the flux of Ca2+ ions within hepatocytes, consequently exerting regulatory effects on hepatic lipid metabolism (42). Therefore, folate’s modulation of fatty acid metabolism in hepatocytes may be associated with its ability to ameliorate mitochondrial-ER functionality and enhance their interorganellar crosstalk. While direct evidence of folate’s role in regulating MAMs is lacking, its simultaneous actions on mitochondria and ER raise the possibility that it may help preserve MAM homeostasis. Future investigations are needed to clarify this mechanism and to determine whether MAMs represent a therapeutic target for folate intervention in AFLD. Furthermore, betaine, which shares a functional relationship with folic acid in methyl metabolism, can promote the methylation of phosphatidylethanolamine (PE) to phosphatidylcholine (PC). This action facilitates VLDL synthesis and the export of triglycerides (TG) out of cells, thereby reducing intracellular TG accumulation (43). Thus, the beneficial effects of folic acid on AFLD may involve not only the regulation of hepatic lipogenesis but also the enhancement of lipid transport and metabolism.

We investigated the role of folic acid in SREBP-1c-mediated lipogenesis in AFLD; however, there are still a few limitations to be aware of. First, although our findings were supported by both epidemiological analyses (NHANES data) and experimental models (mice and hepatocytes), the use of animal and *in vitro* models cannot fully replicate the complex pathophysiological processes of alcoholic fatty liver disease (AFLD) in humans. Thus, caution is needed when extrapolating these results directly to clinical populations. Second, although the integration of animal and cellular experiments strengthens the causal interpretation of our findings, analyses based on the NHANES database remain subject to unavoidable residual confounding. Despite careful adjustment for multiple covariates, unmeasured factors such as detailed dietary patterns, genetic polymorphisms, or concomitant diseases could still influence the observed associations. Third, the study used a single folic acid dosage in the animal intervention without a dose–response gradient. This may limit the generalizability of our conclusions, as the optimal dose and long-term safety of folic acid supplementation for AFLD prevention remain to be established in future clinical trials. Fourth, the subgroup effect of RBC folate may have potential translational value in identifying high-risk individuals who could benefit from early nutritional intervention. However, practical application would require further steps, including validation of cutoff values in larger and more diverse cohorts, as well as an assessment of feasibility and cost-effectiveness in real-world screening, while more in-depth research is needed before clinical implementation.

## 5 Conclusion

In summary, our findings indicate that serum folate levels act as a protective factor against AFLD. Folic acid supplementation mitigates hepatic TG accumulation by downregulating the SREBP-1c-mediated fatty acid synthesis pathway. These results support folic acid intervention as a potential novel strategy for the prevention and treatment of AFLD.

## Data Availability

The raw data supporting the conclusions of this article will be made available by the authors, without undue reservation.
