# Supplementary material for: Folic acid supplementation ameliorates alcohol-induced hepatic steatosis by inhibiting SREBP-1c-mediated lipogenesis
Source: Front Nutr. 2025 Sep 24;12:1668430. doi: 10.3389/fnut.2025.1668430 (PMC12505660; doi:10.3389/fnut.2025.1668430)
Supplement: Supplementary file 1 [file Supplementary_file_1.docx]

**
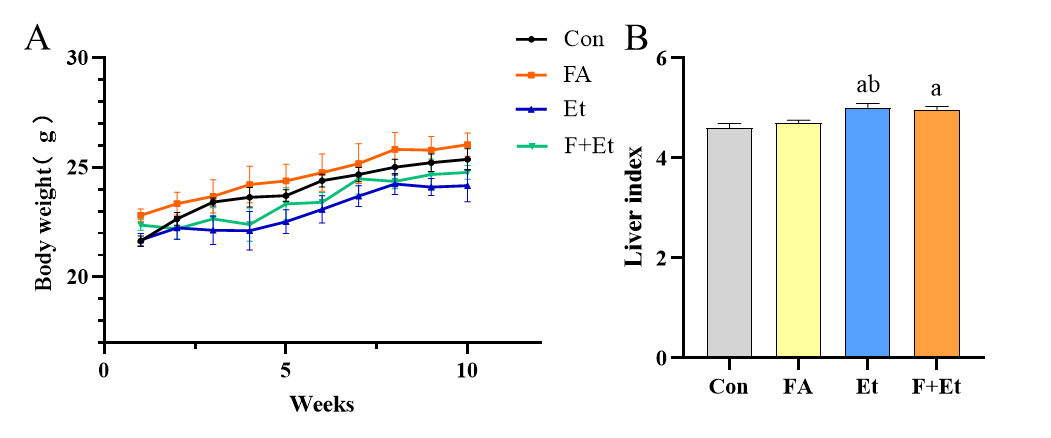
**

**Figure S1.** **The body weight changes among the model construction.** Data are presented as mean ± SEM (n=20/group). A signiﬁcant diﬀerence (*P*<0.05) is identiﬁed by diﬀerent letters: a, vs. the Con group; b, vs. the FA group; c, vs. the Et group; d, vs. the F + Et group.

**Figure S2.** **Serum folate levels for each group are expressed as the mean ± SEM (n=3).** A signiﬁcant diﬀerence (*P*<0.05) is identiﬁed by diﬀerent letters: a, vs. the Con group; b, vs. the FA group; c, vs. the Et group; d, vs. the F + Et group. A total of 0.1mL of serum for each sample was diluted with distilled water at a 1:9 dilution and then centrifuged at 4 ℃ 10000 r/min for 10 min (such process should protect from light during the whole operation), and take 20 μL supernatant measured by HPLC (Agilent 1260, with a 280 nm diode-array detector and cementation workstation, mobile phase: 0.2% phosphoric acid water: acetonitrile = 90:10, velocity: 1 mL/min).
